# Navigating the 2021 update to the IDSA/SHEA *Clostridioides difficile* guidelines: An ethical approach to equitable patient care

**DOI:** 10.1017/ash.2022.49

**Published:** 2022-04-25

**Authors:** Christopher J. Shoff, S. Shaefer Spires, Christina Yen, Sonali D. Advani

**Affiliations:** 1 Division of Infectious Diseases, Department of Medicine, Duke University School of Medicine, Durham, North Carolina; 2 Duke Center for Antimicrobial Stewardship and Infection Prevention, Durham, North Carolina; 3 Division of Infectious Diseases, University of Texas Southwestern School of Medicine, Dallas Texas

## Abstract

The 2021 focused update to the Infections Diseases Society of America/Society for Healthcare Epidemiology of America (IDSA/SHEA) guidelines for management of *Clostridioides difficile* infection (CDI) prioritizes the use of fidaxomicin over vancomycin for the treatment of initial and recurrent CDI. These recommendations have significant clinical and financial ramifications for hospitals and patients with CDI. Antimicrobial stewardship programs must balance the needs, goals, and barriers faced by patients and health systems when determining the best treatment strategy for CDI. In this commentary, we provide antimicrobial stewardship programs with a decision-making framework that acknowledges the fundamental principles of ethics to provide equitable patient care.

*Clostridioides difficile* infection (CDI) places a significant burden on the US healthcare system. The Centers for Disease Control and Prevention estimated a national burden of 462,100 cases annually with an incidence of 143.6 per 100,000 population.^1^ In 2015, the estimated cost of total inpatient CDI-associated care eclipsed US$6 billion, with a presumed total financial cost greater than US$12 billion.^2^ The burden of CDI on individual patients is also severe. Despite changing treatment strategies and emerging therapeutics, overall risk of recurrence remains constant at ∼20%. Furthermore, CDI prolongs hospitalizations, and recent evidence suggests that recurrent and more severe disease are associated with lower quality of life.^3^

In 2021, the Infectious Diseases Society of America (IDSA), in collaboration with the Society for Healthcare Epidemiology of America (SHEA), published a focused update with specific recommendations for the treatment of initial CDI, recurrent CDI, and the role of bezlotoxumab.^4^ In this article, we review the major recommendation updates, highlight the ethical and clinical dilemmas arising from them, and suggest a principlist approach to addressing these recommendations on individual, programmatic, and healthcare system levels.

## Changes to recommendations in the 2021 focused update

Three major changes appear in the updated CDI guidelines (Table 1). The change in treatment recommendation for initial CDI is based upon pooled data from 4 clinical trials.^5–8^ In the pooled analysis, fidaxomicin improved sustained response at 4 weeks, when compared to a 10-day regimen of vancomycin. No differences in cure rates, mortality, or adverse events were detected, but there was a decrease in recurrence with fidaxomicin. For recurrent episodes of CDI, the new recommendation results from a pooled subgroup analysis of 3 randomized clinical trials^5–7^ demonstrating that fidaxomicin improved sustained response at 30 days. However, this response was not sustained through the 90-day follow-up. Additionally, cure rates, mortality, and adverse events were comparable between treatment with fidaxomicin and vancomycin. Finally, the new guidelines address the use of bezlotoxumab, which has not been previously addressed. The recommendation for bezlotoxumab results from a pooled analysis of 2 clinical trials^9,10^ demonstrating that cotreatment with bezlotoxumab reduced both CDI recurrence at 12 weeks and CDI-associated, 30-day hospital readmission. Notably, <5% of patients in the analysis for bezlotoxumab received cotreatment with fidaxomicin; thus, it remains unclear whether the benefit of bezlotoxumab is conserved in patients receiving fidaxomicin.

**Table 1.** Focused Treatment Updates to the IDSA/SHEA *Clostridioides difficile* Infection Guidelines

CDI Episode	2017 IDSA/SHEA CDI Guidelines	2021 IDSA/SHEA CDI Update
Initial CDI episode	Oral vancomycin or fidaxomicin, as an alternate, for treatment of first episodes	Fidaxomicin preferred over standard course of oral vancomycin for first cases of CDI
Recurrent CDI episode	Pulsed and tapered vancomycin for treatment, with fidaxomicin as an acceptable alternative	Fidaxomicin as either continuous or pulse therapy recommended over standard course of oral vancomycin for cases of recurrent CDI
Adjunctive use of bezlotoxumab	Not addressed	Bezlotoxumab recommended as adjunctive therapy for CDI recurring within 6 mo and may also be considered for initial CDI, where recurrence risk is high.^a^

Note. CDI, *Clostridioides difficile* infection.

a High risk is defined as the presence of 1 or more of the following risk factors: age >65 years, immunocompromise, or severe CDI on presentation.

## Concerns with recommendation changes

As discussed above, the change in recommendations result from pooled analyses and subgroup analyses, which demonstrate modest benefit of fidaxomicin over vancomycin regarding recurrent disease, without any benefit with respect to clinical cure or mortality. The panel’s heavy emphasis on the avoidance of recurrent CDI is supported by data suggesting that patients with recurrent disease suffer from poorer quality of life.^3^

However, several counterarguments can be made with respect to the new recommendations, including increased financial consequences to the patient and health system. Regarding increased costs to the individual, a recent study of Medicare enrollees showed that only 1.1% had access to fidaxomicin and 14.4% had access to vancomycin as a tier 1 or tier 2 medication. Using current pricing, this amounted to a >$1,500 out-of-pocket cost for a 10-day course of fidaxomicin.^11^ Many patients cannot afford such costs for medical care; a recent survey found 56% of Americans could not cover a surprise $1,000 expense.^12^ Financial costs may be incurred by the healthcare system. In 2 randomized, phase 3 clinical trials, fidaxomicin was noninferior to vancomycin for clinical cure (88.2% vs 85.8%; 91.7% vs 90.6%, respectively) and led to fewer recurrences (15.4% vs 25.3%; 12.7% vs 26.9%, respectively). With a lower recurrence rate of ∼10% with fidaxomicin versus vancomycin, it would cost ∼$20,000 (number needed to treat, 10) to prevent 1 recurrence.^13^ Although several studies have reported the cost-effectiveness of fidaxomicin for treatment of CDI, many of these studies were industry funded or used European drug pricing for modeling, in which the cost of fidaxomicin is almost half the cost in the United States.^14–16^ Although a newer, non–industry-funded study also suggested the cost-effectiveness of fidaxomicin, the model was highly dependent on hospital length of stay.^17^ It is not difficult to imagine a scenario in which a hospitalized patient is started on fidaxomicin, but discharge is delayed when it is discovered that the patient cannot afford the outpatient cost of fidaxomicin. In such a case, prolonging hospitalization for financial and logistical reasons may aggravate financial and emotional strain on patients. Alternatively, a hospitalized patient discharged on fidaxomicin, may find that they cannot afford their prescription, and thus may fail to complete a full course of therapy, potentially increasing recurrence risk. Finally, it remains unclear whether the initial purchasing cost of fidaxomicin will have to be covered by the healthcare system or whether insurers will support the use of these agents. It seems entirely plausible that excess purchasing cost may be offset from antimicrobial stewardship budgets, leaving less economic freedom for other ventures in stewardship.

## An ethical framework for discussions

In medicine, ethical dilemmas are frequently adjudicated by Beauchamp and Childress’ 4 principles: respect for autonomy, beneficence, nonmaleficence and justice.^18^ Respect for autonomy values the patient’s right for self-determination. Beneficence describes promoting good, while nonmaleficence aims to prevent harm. Finally, justice ensures equity and fairness.

Each principle must be balanced against the others, with one or more principles prioritized over the others in certain scenarios. In the following sections, we offer some perspective on the 2021 focused update with respect to each of these tenets. We review the role of each principle in the application of the updated recommendations to individual patient–physician discussion, antimicrobial stewardship program recommendations, and healthcare-system considerations. Finally, we pose questions and considerations related to each principle that clinicians, stewards, and health systems should contemplate when implementing the updated guidelines.

### Respect for autonomy

Respect for autonomy is best described as an individual’s right to determine his or her own medical decision making. Although autonomy is often listed first among the ethical principles, it is important to recognize that autonomy does not supersede the other 3 principles. Physicians must ensure that patients make informed decisions without undermining the physician’s responsibility to promote beneficial and equitable choices. As such, when treating patients with CDI, the clinician should highlight the decreased recurrence risk with fidaxomicin over vancomycin, but also disclose that other potentially equally valuable patient outcomes, such as death, adverse events, and cure rates are equivalent between treatments. It is also important for the treating physician to disclose the potentially increased out-of-pocket cost of fidaxomicin and to understand whether the information regarding increased cost is perceived as an impediment or empowering knowledge. Armed with this information, 2 different patients, or even the same patient at different points in time, may choose different treatment options. For example, one patient battling recurrent CDI may prioritize the clinical benefit that fidaxomicin offers over vancomycin, and choose a treatment associated with higher out-of-pocket costs, whereas a different patient with a first episode of CDI may value the economic advantage of vancomycin instead. Neither is wrong; both are making informed, autonomous decisions based on an ethically informed discussion with their physician.

Antimicrobial stewardship program staff, healthcare system leadership, and pharmacy and therapeutics committees must self-reflect when considering the addition of fidaxomicin to their institutional guidelines and formularies. Discussing cost is unavoidable when balancing the patient’s right to select a medication against the institution’s ability to provide it. However, a candid consideration of these questions during the preimplementation stage will permit stewards and systems to respond to these requests. One potential solution is to partner with specialty pharmacies to create a referral network with other hospitals with access to fidaxomicin. If fidaxomicin in inaccessible or restricted at the health-system level, this decision should be reviewed in multidisciplinary meeting with all key stakeholders and defined in an institutional policy so the treating physician does not bear the responsibility for limiting a patient’s choice.

### Beneficence and nonmaleficence

In the Hippocratic Oath, the phrase primum non nocere (first, do no harm) summarizes nonmaleficence, whereas beneficence goes one step further—acting to optimize benefit. When we discuss benefit and harm, are we beholden only to the patient’s clinical outcome, or should other outcomes, such as financial and social well-being be considered too? From a purely medical perspective, maximizing beneficence and minimizing harm supports fidaxomicin, given its superiority over vancomycin regarding recurrence rates, and similar adverse effect profile. The guideline authors explicitly acknowledge this by stating “this recommendation places a high value in the beneficial effects and safety of fidaxomicin.”^4^

However, patient harm may occur by undervaluing the challenges posed by fidaxomicin’s cost and availability. Currently, most guidelines do not account for the harms incurred by patients as a result of medication cost, or they give it less weight with respect to clinical outcomes.^19^ Cost-effectiveness studies attempt to factor cost into medical decision making; however, the results of these studies are highly dependent on a priori assumptions for model inputs, and they are often performed from the perspective of the healthcare system and not the individual patient. This situation makes it challenging for physicians and patients to predict how financial harm could result in clinical harms. Patients could be forced to choose between paying for fidaxomicin versus other medications and necessities, which could exacerbate other comorbidities or cause transportation, housing, or food insecurity, which could influence routine medical care. Conversely, failing to prescribe fidaxomicin could result in costly hospitalization for CDI recurrence.

Finally, some pharmacies, stewardship programs, and healthcare systems may be unable to justify the cost of maintaining fidaxomicin on formulary, limiting its access to patients that meet specific criteria. Cost-effectiveness, healthcare outcomes, and implementation studies are needed to understand the impact of access limitations when caring for patients with CDI. Policies should be developed to protect patients who and healthcare systems that choose to assume this expense.

### Justice

Justice in this context may be summarized by the statement, “All patients with similar cases of CDI should have equitable access to equivalent therapies.” However, due to variable copays and varying patient financial means, equitable financial access does not exist. Currently, fidaxomicin remains significantly more expensive when compared to oral vancomycin, which the focused update to the guidelines acknowledge by stating, “Implementing this recommendation [for fidaxomicin over vancomycin] probably reduces equity due to variation in medical insurance coverage.”^4^ By prioritizing beneficence, justice was deprioritized, as remarked upon in the assessment that “Resource use (monetary costs and cost-effectiveness, for example) was rated as ‘of limited importance.’”^4^

Furthermore, pursuing justice also implies a desire to ensure equitable availability. Drugs are not often uniformly distributed at the local, national, and global level. Many pharmacies may not carry fidaxomicin, either secondary to its significant cost or the relative infrequency with which it is prescribed. How can prescribers or institutions adhere to guideline-based practice when many patients simply cannot access fidaxomicin? How can patients access fidaxomicin when their physicians may not have access?

Framing the challenge of therapeutic equity using these 2 questions, prescribers can invoke the guideline’s designation of oral vancomycin as an acceptable alternative to fidaxomicin. If oral vancomycin is a less acceptable alternative, given a patient’s needs and values, disclosing the limitations of access to fidaxomicin not only preserves the patient–physician relationship but also allows for exploration of ways in which a patient might obtain such access. Increased work by patient advocacy groups and greater public awareness are necessary to improve access to preferred medications.

Justice is not simply achieved by adding fidaxomicin to a formulary. We suggest that health systems, pharmacy and therapeutics committees, and antimicrobial stewardship programs make efforts to mitigate patient financial inaccessibility to fidaxomicin. If eliminating financial barriers is not possible, then entities wishing to maximum “fairness” should consider utilizing oral vancomycin in favor over fidaxomicin. Additionally, patients should receive support for accessing fidaxomicin, including strategies such as maintaining a list of local or regional pharmacies with supply, or partnership with mail-order pharmacies and patient assistance programs.

## Conclusion

In conclusion, the implementation of recommendations in the 2021 focused-treatment update to the IDSA/SHEA *Clostridioides difficile* guidelines may be challenging for health systems, prescribers, and patients. Although fidaxomicin has demonstrated a more sustained response compared with standard of care (ie, vancomycin only), it has not been shown to significantly reduce mortality or adverse drug reactions or to achieve faster clinical cure.^5,7,8^

**Fig. 1.**
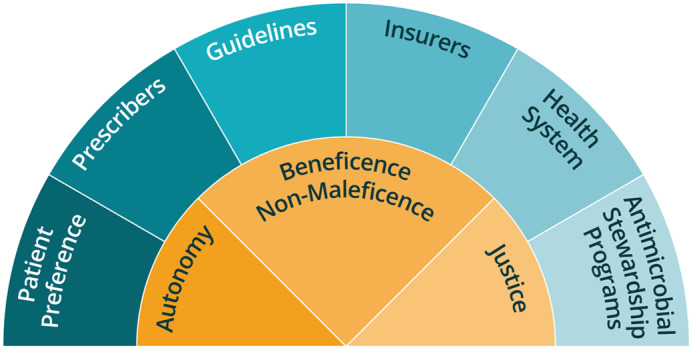
The interplay between ethical principles and healthcare entities and considerations.

Sustained response was also not statistically improved in the 90-day follow-up for recurrent infections, reducing the clinical significance of the 30-day follow-up outcome.^4^ Furthermore, data delineating which patient populations may benefit from one therapy over the other and where vancomycin tapers are clinically useful are limited. Finally, real-world barriers related to hospital costs, patient costs, and insurance coverage, significantly diminish the applicability of this recommendation. When considering the optimal treatment regimen, prescribers, antimicrobial stewardship programs, and health systems should consider their individual priorities and risks for patients, and they should utilize the principles of autonomy, beneficence, nonmaleficence, and justice to inform their treatment decisions (Fig. 1). Some entities—patients, prescribers, or health systems—may emphasize 1 or more principles that may influence treatment practice. A balanced, blended approach considering all 4 of these principles will often result in equitable patient care.
